# Nœud intracardiaque du cathéter de Swan-Ganz: à propos d'un cas

**DOI:** 10.11604/pamj.2013.14.137.1635

**Published:** 2013-04-08

**Authors:** Heithem Chemchik, Mohamed Ben Hassen, Mohamed Turki, Ghazi Aissaoui, Karim Gahbiche, Walid Naija, Imen Mgarrech, Chokri Kortas, Rachid Said

**Affiliations:** 1 Service d'anesthésie-réanimation CHU Sahloul de Sousse,Tunisie; 2 Service de chirurgie cardiovasculaire et thoracique CHU Sahloul de Sousse, Tunisie

**Keywords:** Cathéter, Swan-Ganz, complication, Nœud, catheter, Swan-Ganz, complication, node

## Abstract

Le monitorage hémodynamique par cathéter de Swan-Ganz est utile surtout en cas de fonction cardiaque altérée. La mise en place de ce cathéter peut être accompagnée par des complications sévères dans 3 à 4,4% des cas tel que la rupture de l'artère pulmonaire, l'atteinte valvulaire, les troubles de la conduction, le pneumothorax, l'hémothorax et rarement la formation d'un nœud intracardiaque. Nous rapportons un cas de cathéter de Swan-Ganz compliqué d'un nœud formé à son extrémité et nous discutons les éventuels moyens diagnostiques et thérapeutiques de cette complication.

## Introduction

Le cathéter de Swan-Ganz reste le moyen de référence pour la mesure du débit cardiaque. Il permet la mesure des pressions du remplissage du cœur droit et gauche. Son indication doit être raisonnée et relève de la compétence médicale. La mise en place de ce cathéter peut être accompagnée par plusieurs types de complication telle que l'apparition d'un nœud à son extrémité distale. Nous rapportons un cas de cathéter de Swan-Ganz compliqué d'un nœud formé à son extrémité et nous discutons les éventuels moyens diagnostiques et thérapeutiques de cette complication.

## Patient et observation

Un patient âgé de 59 ans ayant présenté un infarctus de myocarde au niveau du territoire inférieur avec à la coronarographie une sténose serrée du tronc commun gauche moyen et distal, une sténose serrée de l'inter-ventriculaire antérieure (IVA), une circonflexe occluse après la naissance d'une belle branche marginale et une sténose serrée de la coronaire droite. Ce patient a été programmé pour pontage coronaire, par montage en Y des artères mammaires internes gauche et droite, de l'IVA et de la première marginale. L’échographie cardiaque trans-thoracique préopératoire a montré une cardiomyopathie ischémique à fonction ventricule gauche altérée avec une fraction d’éjection estimée à 28%, une dilatation ventriculaire droite et une hypertension artérielle pulmonaire (HTAP) à 46mmHg. Le bilan biologique préopératoire était correct. La radiographie thoracique a montré une cardiomégalie et un épanchement pleural liquidien bilatéral de faible abondance. L’électrocardiogramme a montré un rythme régulier sinusal à 90 cycles/min, une onde Q en antéro-septo-apical et un bloc de branche gauche incomplet.

A son arrivée au bloc opératoire, un monitorage de la fréquence cardiaque, de la saturation artérielle en oxygène, de la pression artérielle invasive et de la pression veineuse centrale (PVC) ont été mis en place. Une anesthésie générale a été pratiquée avec une induction faite par 20mg d'Etomidate, 500’ de Fentanyl et 12mg de Cis-atracurium. L'entretien anesthésique a été fait par Fentanyl, Cis-atracrium et Sevoflurane. Un cathéter de Swan-Ganz, 7 French 110 cm 4-lumen type Baxter, a été mis en place au niveau jugulaire interne droit pour affiner le monitorage hémodynamique. Sa mise en place a été marquée par la difficulté de son passage du ventricule droit vers l'artère pulmonaire. La courbe de la pression artérielle pulmonaire avait un aspect normal et elle a confirmée l'HTAP mentionnée à l’échographie cardiaque préopératoire. Par contre le gonflage du ballonnet était difficile à réaliser avec impossibilité de mesurer la pression au niveau des capillaires pulmonaires (PCP).

**Figure 1:**
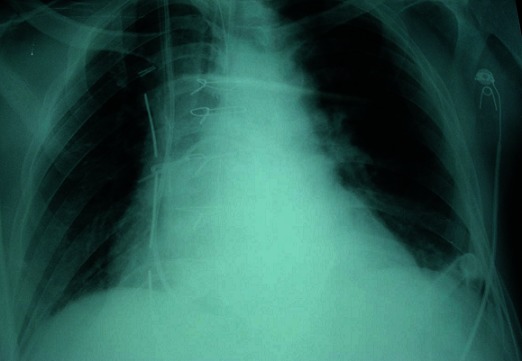
Nœud du cathéter de Swan-Ganz dans l'artère pulmonaire droite

**Figure 2:**
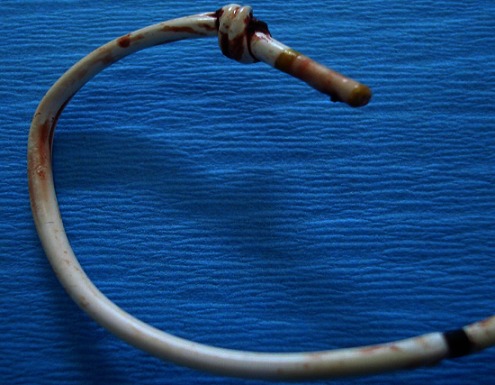
Un nœud simple à 14 mm de l'extrémité distale du cathéter de Swan-Ganz

En post opératoire, une radiographie du thorax a été faite montrant un nœud du cathéter de Swan-Ganz au niveau de l'artère pulmonaire (Figure 1). Une ouverture du nœud par un cathéter percutané par voie fémorale sous contrôle radiologique a échoué. La conduite à tenir était le recours à la chirurgie avec cervicotomie sous bloc cervical. L'ouverture de la veine jugulaire interne droite a montré un nœud simple et serré à 14mm de l'extrémité distale du cathéter (Figure 2).

## Discussion

Les complications du cathéter de Swan-Ganz peuvent être graves tels que la rupture de l'artère pulmonaire, l'atteinte valvulaire, les troubles de la conduction, le pneumothorax, l'hémothorax et rarement la formation d'un nœud intracardiaque [1]. L'incidence de cette dernière complication n'a pas été évaluée. La revue de la littérature montre que le premier cas était décrit par Johanson en 1954 [2]. Dans l’étude de Bossert incluant 3730 cathéters de Swan-Ganz, le nœud sur cathéter de Swan-Ganz n'a été observé que dans un seul cas. Il peut être favorisé par plusieurs facteurs: la dilatation du ventricule droit, l'insertion de la sonde à un niveau supérieur à 50 cm, le passage du ventricule droit vers l'artère pulmonaire avec un ballonnet qui n'est pas bien gonflé [3, 4]. L'impossibilité de mesurer la pression capillaire pulmonaire doit faire suspecter un nœud sur le cathéter. La radiographie standard est suffisante pour confirmer ce diagnostic. Dans la littérature, la conduite à tenir n'est pas univoque. La meilleure technique et la moins invasive, utilisée si le nœud n'est pas trop serré, consiste à introduire un guide métallique à travers la lumière du cathéter sous contrôle radiologique permettant de libérer le nœud. Cette technique peut échouer quand le nœud n'est pas assez lâche et/ou quand il est situé à une distance lointaine de la pointe du cathéter [1]. Une autre technique possible est la réduction du diamètre du nœud, par la traction de ce dernier contre une gaine veineuse 8F introduite à travers l'extrémité proximale du cathéter, permettant de retirer le cathéter par la veine jugulaire interne [1]. La traction puis l'enlèvement du cathéter à travers le site de ponction jugulaire interne n'est pas une procédure sûre puisqu'elle pourrait provoquer des lacérations veineuses. L'abord chirurgical de la veine est plus garanti. C′était la procédure de choix dans notre cas. La technique la plus décrite est l'extraction du nœud par un cathéter percutané par voie fémorale sous contrôle radiologique [5, 6]. La chirurgie à cœur ouvert est réservée en cas d’échec de la radiologie interventionnelle.

## Conclusion

La formation d'un nœud au niveau d'un cathéter de Swan-Ganz est une complication extrêmement rare. Elle doit être évoquée devant la difficulté de mesure de la pression des capillaires pulmonaires. L'impossibilité de retrait de cathéter impose l'ouverture du nœud par radiologie interventionnelle ou son retrait par voie chirurgicale.

## References

[1] Lopes MC, de Cleva R, Zilberstein B, Gama-Rodrigues JJ (2004). Pulmonary artery catheter complications: report on a case of a knot accident and literature review. Rev Hosp Clin Fac Med Sao Paulo.

[2] Johansson L, Malmstrom G, Uggla LG (1954). Intracardiac knotting of the catheter heart catheterization. J Thorac Surg.

[3] Jagers op Akkerhuis M, Bauland CG, Voets AJ (1999). Percutaneous removal of a knotted pulmonary artery catheter using a tracheostomy dilator. Crit Care..

[4] Ranatunga DG, Richardson MG, Brooks DM (2007). Percutaneous fluoroscopic removal of a knotted Swan-Ganz catheter in a patient with a persistent left-sided superior vena cava. Australas Radiol..

[5] Katsikis A, Karavolias G, Voudris V (2009). Transfemoral percutaneous removal of a knotted Swan-Ganz catheter. Catheter Cardiovasc Interv.

[6] Rahim SA, Franke R, Mathew V (2009). Removal of a knotted Swan-Ganz catheter. J Am Coll Cardiol..

